# Reducing Back Exertion and Improving Confidence of Individuals with Low Back Pain with a Back Exosuit: A Feasibility Study for Use in BACPAC

**DOI:** 10.1093/pm/pnad003

**Published:** 2023-02-16

**Authors:** D Adam Quirk, Jinwon Chung, Gregory Schiller, Jason M Cherin, Philipp Arens, David A Sherman, Emma R Zeligson, Diane M Dalton, Lou N Awad, Conor J Walsh

**Affiliations:** John A. Paulson School of Engineering and Applied Sciences, Harvard University, Boston, MA, United States; Wyss Institute for Biologically Inspired Engineering, Harvard University, Boston, MA, United States; John A. Paulson School of Engineering and Applied Sciences, Harvard University, Boston, MA, United States; Wyss Institute for Biologically Inspired Engineering, Harvard University, Boston, MA, United States; College of Health & Rehabilitation Sciences: Sargent College, Boston University, Boston, MA, United States; John A. Paulson School of Engineering and Applied Sciences, Harvard University, Boston, MA, United States; John A. Paulson School of Engineering and Applied Sciences, Harvard University, Boston, MA, United States; John A. Paulson School of Engineering and Applied Sciences, Harvard University, Boston, MA, United States; College of Health & Rehabilitation Sciences: Sargent College, Boston University, Boston, MA, United States; College of Health & Rehabilitation Sciences: Sargent College, Boston University, Boston, MA, United States; College of Health & Rehabilitation Sciences: Sargent College, Boston University, Boston, MA, United States; Wyss Institute for Biologically Inspired Engineering, Harvard University, Boston, MA, United States; College of Health & Rehabilitation Sciences: Sargent College, Boston University, Boston, MA, United States; John A. Paulson School of Engineering and Applied Sciences, Harvard University, Boston, MA, United States; Wyss Institute for Biologically Inspired Engineering, Harvard University, Boston, MA, United States

**Keywords:** low back pain, biomechanics, exoskeletons, electromyography, kinesiophobia

## Abstract

**Objective:**

Low back pain (LBP) is hallmarked by activity limitations, especially for tasks involving bending. Back exosuit technology reduces low back discomfort and improves self-efficacy of individuals with LBP during bending and lifting tasks. However, the biomechanical efficacy of these devices in individuals with LBP is unknown. This study sought to determine biomechanical and perceptual effects of a soft active back exosuit designed to assist individuals with LBP sagittal plane bending. To understand patient-reported usability and use cases for this device.

**Methods:**

Fifteen individuals with LBP performed two experimental lifting blocks once with and without an exosuit. Trunk biomechanics were measured by muscle activation amplitudes, and whole-body kinematics and kinetics. To evaluate device perception, participants rated task effort, low back discomfort, and their level of concern completing daily activities.

**Results:**

The back exosuit reduced peak back extensor: moments by 9%, and muscle amplitudes by 16% when lifting. There were no changes in abdominal co-activation and small reductions maximum trunk flexion compared to lifting without an exosuit. Participants reported lower task effort, back discomfort, and concern about bending and lifting with an exosuit compared to without.

**Conclusions:**

This study demonstrates a back exosuit not only imparts perceptual benefits of reduced task effort, discomfort, and increased confidence in individuals with LBP but that it achieves these benefits through measurable biomechanical reductions in back extensor effort. The combined effect of these benefits implies back exosuits might be a potential therapeutic aid to augment physical therapy, exercises, or daily activities.

## Introduction

Low back pain (LBP) is among the world’s leading causes of disability with global socio-economic implications.^1^ In the United States, LBP is the single most expensive health condition, with growth outpacing overall healthcare spending due to largely ineffective medical treatments.^2^ While most LBP cases have no clear pathoanatomical cause,^3^ a contributor to LBP development is the amount and intensity of spinal loading.^4^ Once LBP arises, individuals' disability experience can be variable, ranging from the difficulty of performing domestic chores to loss of work with common limitations stemming from an inability to bend, squat, or stoop.^5^

Adding to this disability, individuals with LBP tend to believe biomechanical factors are the cause of their back pain,^6^ which could cause flare-ups.^7^ For some, this belief can lead to fear of provocative movements resulting in movement avoidance and postural adaptations.^8^ Specifically, individuals with LBP naturally select biomechanical compensations that reduce loads and guard against pain and instability.^9^^,^^10^ Such compensatory patterns, however, are not proven to be efficacious for recovery, as slower and stiffer biomechanics result in lower spinal range of motion (RoM) and greater trunk muscle activity over time.^11–13^ Moreover, the resulting lifestyle adjustments can further exacerbate the problem and ultimately lead to structural pathologies and pain persistence.^14^

To prevent these compensatory behaviors, ergonomic aids and orthotic devices have been explored to help bridge function during periods of low-back-related disability. Back belts, for instance, aim to stiffen the spine and increase abdominal pressure, thereby increasing spinal stability. However, they have shown minimal biomechanical efficacy,^15^ restricting RoM^16^ and disrupting inter-segmental coordination.^17^ Despite limited biomechanical efficacy, back belts transiently improve pain, fear of movement, and catastrophizing in people returning to work following LBP,^16^ highlighting the psychological implications of perceived support.

Emerging robotic technologies, such as back exoskeletons or exosuits (herein referred to as back exos), have been discussed as a potential aid in rehabilitation by assisting natural biomechanics while offloading back exertion with external assistance.^18^ Back exos can be classified as active, powered by electrical, hydraulic, or pneumatic actuators, or passive, unpowered devices that use a spring-like structure to store and release energy collected by human motion.^19^ A number of studies in healthy participants have demonstrated that both passive and active back exos can reduce peak muscle activity through these applied external moments, which alludes to the potential of these technologies to benefit individuals with LBP.^18^^,^^20^^,^^21^ On the other hand, a few studies with passive exoskeletons have shown that healthy subjects receive these benefits with the burden of increased abdominal coactivation,^18^ movement restriction,^22–24^ and discomfort^23^ during lifting and static bending, which could decrease the likelihood of using back exos with individuals with LBP.^24^

Recently, the effects of passive back exos have been investigated on individuals with mild to moderate LBP.^25–27^ Participants were found to have improved function, reduced task effort, and reduced low back discomfort during lifting and static forward bending while wearing the exoskeleton.^25^ Yasunaga et al. demonstrated that following back exoskeleton therapy, participants had reduced pain and were willing to flex their trunk and hip further.^28^ Despite these benefits, there is growing awareness of the barriers to using back exos for individuals with LBP. Specifically, studies have reported that task restriction, heavy weight, total body discomfort, general bulk and difficulty of donning/doffing result in lower acceptance of the technology.^19^^,^^25^^,^^29^

Past studies highlight the functional and perceptual benefits of back exo technology for individuals with LBP. While it is speculated that these benefits reflect reduced back extensor efforts, similar to that recorded in healthy participants using exos, the biomechanical efficacy of these devices in individuals with LBP remains unknown. It is critical to determine whether individuals with LBP experience similar improvements in back exertion without maladaptation, such as guarded movement patterns^29^ or increased abdominal coactivation. Coactivation is infrequently reported in healthy populations with back exos;^18^ however, it is known that individuals with LBP utilize increased coactivation when exposed to sudden forces increasing this risk.^30^^,^^31^ This maladaptation may incur detrimental effects, such as high cumulative load on the spine,^12^^,^^32^ or tight motor control^33^ that could exacerbate pain or impede the recovery process.^34^

In this study, we evaluated an active back exosuit in individuals with LBP that was previously evaluated in healthy individuals.^35^ The exosuit is a soft active system which provides minimal movement restriction and adaptively applies assistance to a wearer depending on a task such as lowering, lifting, or walking. The device is lightweight (2.7 kg) and easy to wear (average 36 s donning time). In healthy individuals, the device was shown to reduce peak back extensor activation amplitudes by 18% without abdominal coactivation while performing a complicated, 1-hour-long simulated order picking task, including lifting, carrying, and walking.

The purpose of this study is to determine biomechanical and perceptual effects of the back exosuit during sagittal plane lifting in individuals with mild LBP. An additional purpose was to examine the participants' overall impression of the usability and potential use of the exosuit using a device-specific questionnaire. We hypothesized that, similar to healthy individuals, individuals with LBP would experience biomechanical benefits, including reduced measures of peak back extensor: moments and EMG amplitudes, without increased peak abdominal co-activation or inducing reductions in peak sagittal plane trunk flexion when performing constrained squat and stoop lifting tasks with an exosuit compared to lifting without. Because of these biomechanical benefits, we hypothesized, similar to other studies, participants with LBP would experience perceptual benefits, including a reduction in perceived task effort, back discomfort, and levels of concern when using a back exosuit and when envisioning themselves using an exosuit to perform daily lifting and bending activities.

## Methods

### Participants

Fifteen individuals with LBP were recruited from the local community through collaborative relationships with physical therapists for this single-session pilot study. Individuals included were 18–65 years old and were treated for LBP in the past 3 months. Exclusion criteria included self-reported neuromotor or gait disorders, concurrent neck pain, history of low back surgery, osteoporosis, diagnosis of cancer, or current pregnancy. All participants provided verbal and written informed consent prior to beginning the procedures below as approved by Harvard Medical School’s Internal Review Board (IRB18-0960).

### Exosuit

**Figure 1.**
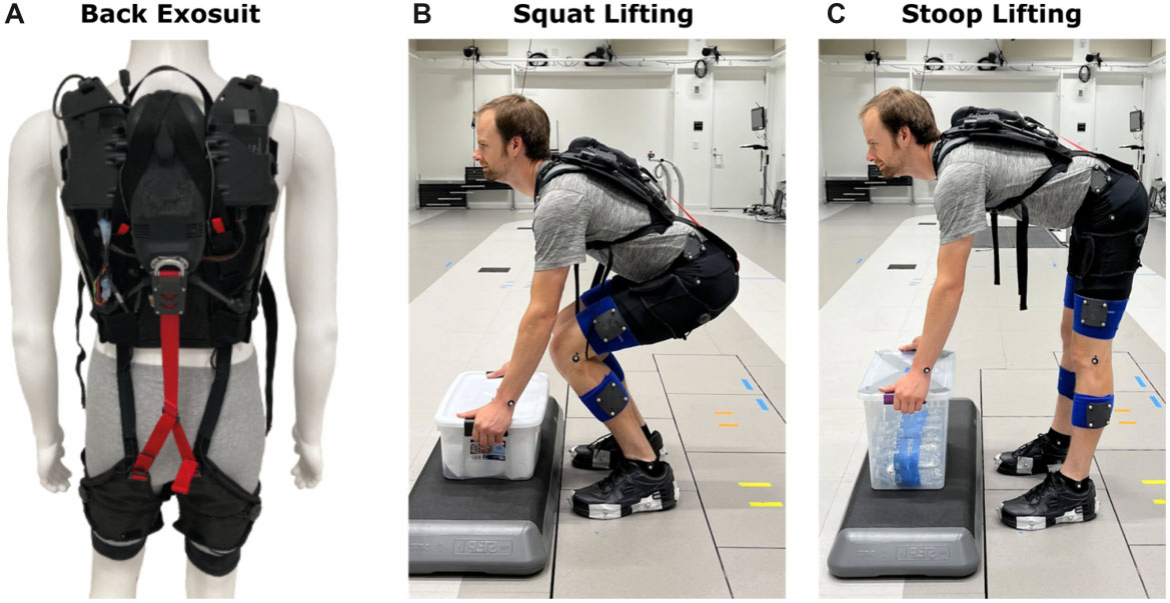
The back exosuit (**A**) used in the study and demonstrated during the squat (**B**) and stoop (**C**) lifting task performed by individuals with LBP with and without the back exosuit displayed in the picture.

Figure 1A shows the soft active back exosuit used in the study. The exosuit utilizes a soft textile-based design to avoid the kinematic constraints associated with rigid mechanisms. A lightweight actuator generates tension on an external ribbon cable spanning the back and hips, using a controller informed by three inertial measurement units (IMUs) designed to measure the torso and the thigh kinematics of the wearer. Overall, the weight of the device is 2.7 kg, including batteries and can deliver up to 30Nm of back extension assistance when set to 100% assistance.

The exosuit assistance is autonomously controlled based on the motion of the wearer with two main principles. First, the exosuit applies assistance proportional to the degree of back and hip flexion of the user. Second, the assistance is scaled based on the movement direction; lower assistance (up to 10Nm) was applied during flexion (lowering) as not to restrict the wearer's RoM and higher assistance (up to 30Nm) was applied during trunk extension (lifting) to offload back exertion.^36^ Through an impedance-based control approach, the exosuit assistance is adaptive to the wearer’s motion rather than forcing them to move in a certain predefined way.^35^

### Protocol

Upon arrival, participants were instrumented for the experiments, including the placement of inertial measurement units (IMU) and electromyography (EMG) sensors. After this, they undertook an informal familiarization session, for approximately 10 minutes. First, comfort with task depth was assessed by asking them to squat toward a box placed at the height of their tibial tuberosity. After confirming they were comfortable with task depth, participants were then taught the squat and stoop lifting task using a 1 kg box, followed by 4 and 6 kg boxes, respectively. A licensed physical therapist was present at each session to ensure safety and comfort with progression. Lastly, participants were introduced to the exosuit. Initially, the exosuit delivered 60% of maximum assistance while participants were encouraged to bend and lift. If comfortable, participants would continue to 80% and 100% assistance.

After suit familiarization, maximum voluntary isometric contraction (MVIC) tasks were conducted. Participants were secured to a HUMAC dynamometer with a peripheral torso adapter (CSMI, Stoughton, MA, United States), and performed one task warm-up, two alternating trunk extension and flexion MVICs. Afterward, participants performed two repetitions of a prone right hip extension MVICs against non-elastic straps. For all contractions, participants were instructed to ramp up for 3 seconds and press into a strap or HUMAC as hard as possible. Participants had two minutes of rest between each repetition.

**Figure 2.**
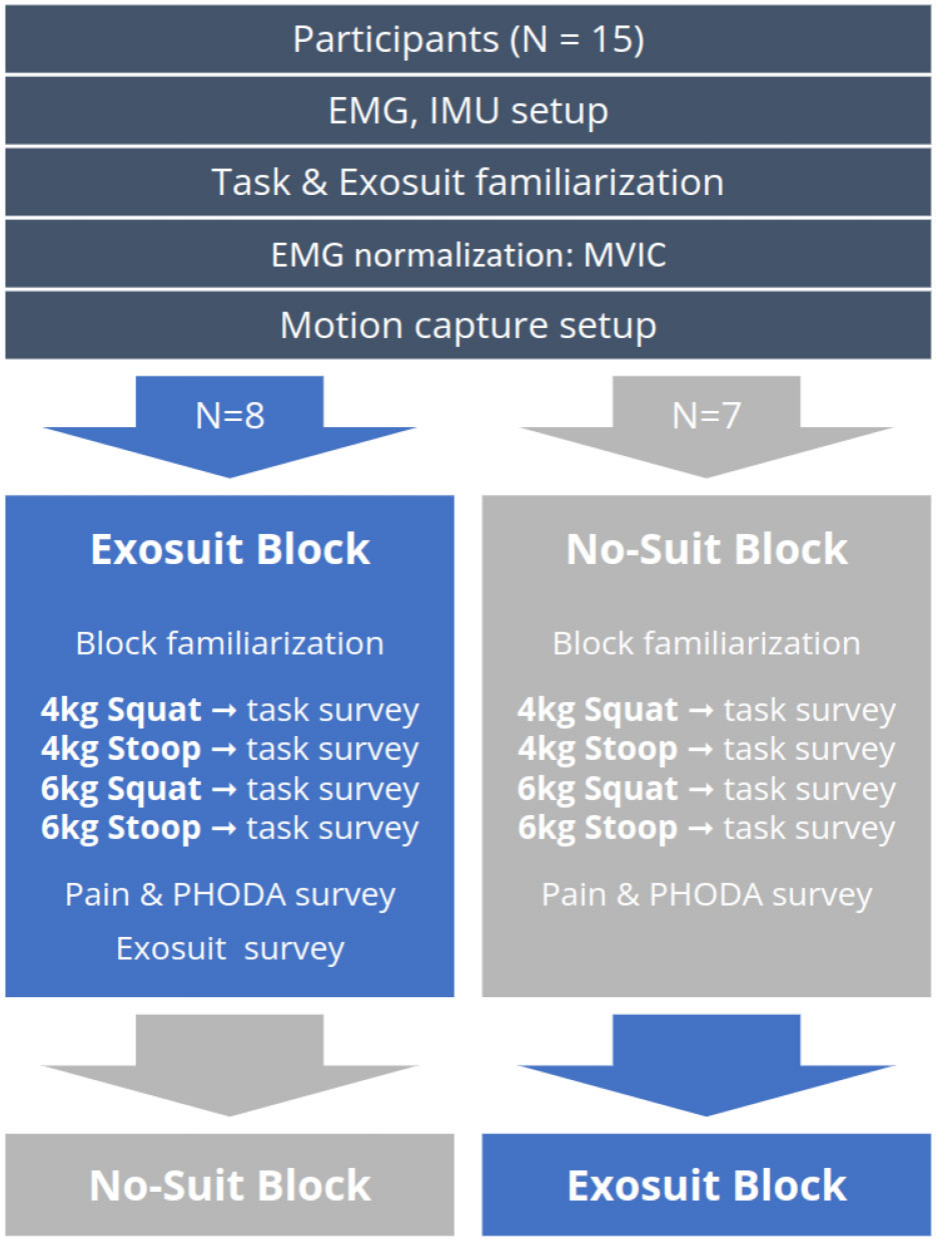
Protocol highlighting the randomization of participants starting with the Exosuit Block or No-Suit Block. Within each block, participants performed four lifting tasks, completing a survey at the end of each task. The end of an experimental block involved completing a pain and photograph series of daily activities (PHODA) surveys and an exosuit-specific survey in the exosuit block only.

Participants were prepared for motion capture, completed a static calibration and transitioned into the two experimental blocks presented using a pseudorandom counterbalanced Latin square approach (Figure 2). Each block started with a brief refamiliarization period allowing participants to become acquainted with the exosuit forces or to wash out exosuit forces from a previous block. These periods consisted of a squat lifting task and a stoop lifting task, with each task consisting of five lifts of a 1 kg box. Following this block familiarization period, participants performed four lifting tasks, two squat lifting tasks and two stoop lifting tasks, with 4 kg and 6 kg boxes. Each task involved five lifts of the designated box, and after each task, participants completed a three-question perceptual survey. Tasks were presented in a constant order that aimed to minimize fatigue (Figure 2).

To limit the confounding influence of changing task dynamics and allow comparison between conditions, our sagittal plane squat and stoop lifting tasks were constrained spatially and temporally. The participants' feet and box position were marked on two separate force plates and the floor respectively, and a repetition would be repeated if the participant noticeably deviated. Lifting was timed to a 50 beats per minute metronome. On “go,” participants would: 1) lower to the box, 2) lift the box to a neutral stand, 3) lower the box to the ground, and 4) return to a neutral stand without the box. The participant then had a brief (7.2 second) rest before their next repetition. For the stoop task, participants “kept their legs as straight as comfortable” as they lifted a 43 × 28 × 32 cm box placed at the height of their tibial tuberosity. In the squat task, participants “bent their knees” to pick up a slightly shorter 46 × 31 × 18 cm (WxLxH) box (Figure 1B and C).

Upon the completion of each task, participants performed a three-question survey. With the completion of an experimental block, participants were given a break to sit down and complete the “end block survey.” Participants would indicate when they were ready to advance to the next task or block and were given the option to skip tasks if needed.

### Data collection setup and processing

#### Inertial measurement units (IMUs) and movement segmentation

Three IMUs (BN0085, BOSCH SensorTec GmbH, Reutlingen, Germany) were positioned on anatomical landmarks and secured to the participant’s skin using adhesives (adhesive spray, double-sided tape, and cover roll tape). A single IMU was positioned on the eighth thoracic spinous process, and one additional sensor was positioned on the posterior aspect of the right thigh, approximately in line with the middle of the gluteal fold and popliteal fossa. For each experimental block, the IMU sensors were directly sampled at 200 Hz using an 8-bit microprocessing unit (PIC18F25K80, Microchip Technology, Inc., AZ, United States) and an onboard flash memory card (SDSQUNC-032G-AN6IA, Scandisk, CA, United States).

Segment angles, directly measured by each IMU sensor were post-processed using custom Matlab code and corrected using a zero-lag 4th order 2 Hz low pass filter for event detection. Movement events were determined by first calculating the relative trunk angle with the subtraction method (T8-Right thigh) around the sagittal plane and deriving angular velocity. The beginning of any constrained lifting repetitions was defined as the time at which trunk flexion velocity exceeds a threshold of 5^°^/s for at least 20 ms (T0). The end of each constrained lifting task repetition was defined as the point at which extension velocity was below 5^°^/s for at least 20 ms (T100). Events were verified using visual inspection and were used to time-normalize all subsequent kinematic, kinetic (exosuit load cell and force plate) and EMG data, which were synchronized using a common signal logged by all equipment.

#### Electromyography (set-up and normalization)

Following standard skin preparation, bar surface electrodes (10 mm interelectrode distance) were positioned over six muscle sites from three muscle groups using standardized guidelines and minor adjustments based on palpation. Muscle sites included for the back extensors: the thoracic (T96) and lumbar erector spinae iliocostalis (L36) (6 cm lateral to the 9th thoracic, and 3rd lumbar spinous process, respectively), and the lumbar longissimus (L13) (3 cm lateral to the 1st lumbar spinous process). For the trunk flexors, signals were monitored from the upper rectus abdominis (URA) (3 cm lateral to the Linea Alba) and middle external obliques (EO) (15 cm lateral to the umbilicus orientated at 45^°^ to the Linea Alba). SENIAM guidelines were used to position the gluteus maximus (GMax). EMG signals were amplified, digitized (2148 Hz), and filtered (Hardware band-pass 20-450Hz) using a series of Duo wireless bioamplifiers and EMGWorks Software (Delsys Inc. Natick, MA, United States).

Custom Matlab (The Math Works^TM^, Natick, MA, United States) code corrected EMG data using a zero-lag 4th order band-pass filter (50–450Hz).^37^ Corrected EMG signals were rectified, and zero-lag 4th order low-pass filtered with a 1 Hz or 6 Hz cutoff for the MVIC tasks or all other tasks, respectively, to produce a linear envelope. Excluding the MVIC tasks, linear-envelope signals were: i) time-normalized (0%–100%) to 101 points using a quadratic spline interpolation algorithm defined by relevant IMU trunk motion events, and ii) amplitude normalized the peak activity captured during the MVIC tasks.^38^ For each constrained lifting task, an ensemble-averaged linear envelope was produced for each muscle site. The primary outcome measure *peak EMG amplitude* was calculated from the ensemble average waveform. Peak EMG was calculated for both the lifting (0%–50%) and lowering (50%–100%) phase of the task. These peak measures were then categorized according to the primary muscle group (back extensor, hip extensor, or trunk flexor) for statistical analysis.

#### Suit kinetics

Tensile force from the exosuit was sampled directly via load cells attached to the active actuator (LSB200, FUTEK Advanced Sensor Technology, Inc., CA, United States), at 200 Hz using an eight-bit microprocessing unit (PIC18F25K80, Microchip Technology, Inc., AZ, United States) and an onboard flash memory card (SDSQUNC-032G-AN6IA, Scandisk, CA, United States). Measured tensile forces were corrected using a zero-lag 4th order low pass filter at 2 Hz in Custom Matlab code. Corrected tensile force data were assumed to be pure acting in the sagittal plane only. These time-varying tensile forces were converted to an extensor moment around the lumbar L5/S1 joint center, by assuming the exosuit acted with a constant moment arm length (MAL) of 0.12 m when considering the flesh margin between PSIS and L5/S1.^39^ However, in many cases this moment arm length was under-estimated when the exosuit ribbon did not make contact with the participant’s pelvis, for example during upright standing. Around the hip; tensile forces were converted to a moment around the hip joint center, first assuming there was a 15% reduction in tensile force when applied to the thigh wrap attributed to friction, and second assuming the hip joint center had a constant MAL of 0.15 m. These lumbar and hip joint moments are referred to as suit moments, which were time normalized from 0% to 100% and utilized in future calculates to measure the biological trunk and hip moment.

#### Whole body kinematics and kinetics

Eleven passive reflective markers were positioned bilaterally on the radial and ulnar styloid, the medial and lateral malleolus, the medial and lateral femoral epicondyles, the greater trochanter, the acromion and the anterior and posterior-anterior iliac spine. Individual passive reflective markers were positioned on the suprasternal notch and the 7th cervical spinous process. Four marker bilateral rigid body clusters were positioned on the iliac crest, thigh, and shank. Following setup, a standing calibration (T-Pose) captured the three-dimensional marker position relative to the rigid bodies using sixteen infrared emitting cameras (Oqus 700, Qualisys) sampled at 200 Hz using Qualisys Track Manager (Version 2020.2, Qualisys^TM^, Goteborg, Sweden). Following calibration, participants performed the constrained movement tasks. For the constrained lifting tasks, ground reaction forces were measured separately from the participants left and right foot positioned on a force plate (Bertec^TM^) sampled at 200 Hz at ±5 V using a 16 bit analog-to-digital board (230599, Qualisys^TM^) in Qualisys Track Manager.

Kinematic and kinetic (force plate) data were post-processed in Visual3D (CMotion Inc., Kingston, ON). All kinematic and kinetic data were 4th order low-pass filtered at 6 Hz. Three-dimensional relative Euler angular kinematics were calculated around the ankle, knee, hip and torso using a flexion-extension, ab-adduction and axial-rotation rotation sequence. For the constrained lifting tasks, a bottom-up inverse-dynamics approach was used to calculate overall moments acting around the ankle, knee, hip, and trunk using a series of Newton-Euler equations assuming each body segment acts as a rigid body. Using this bottom-up approach that terminated at the lumbar joint center, the mass of the exosuit was measured by the force plate. Given the minimal mass (150 g) of each thigh wrap, relevant inertial properties of the exosuit were not modelled in the exosuit condition. All relative angular kinematics and inverse dynamic moments in the sagittal plane were time normalized from 0% to 100% using a quadratic spline interpolation algorithm defined by relevant IMU trunk motion events. Time normalized waveforms were averaged across all repetitions to produce an ensemble average for a specific task. However, inverse-represents dynamics represents the overall or net moment of a joint, without consideration of the external moments produced by an exosuit. Thus, for constrained tasks within the exosuit condition, the primary outcome measure of *biological hip and lumbar moment* was calculated by subtracting suit moment from the sagittal plane overall moment in custom Matlab Script.

The outcome measure for kinetics included both the overall and biological moments for the hip and trunk. Kinematic outcome measures included the sagittal ankle, knee, hip, and trunk angles. Ensemble average kinematic and kinetic waveforms were used to calculate the respective primary outcome measures. For kinematics, the primary outcome measure was the maximum and minimum sagittal plane angle through the entire lifting cycle (0%–100%). For kinetic data outcome measures, peak overall and biological back and hip extensor moments were calculated for both the lifting (0%–50%) and lowering (50%–100%) phases of the task.

#### Perceptual surveys

All perceptual data were collected using a Qualtrics Survey presented on a tablet (Qualtrics XM, Provo, UT, United States). The study had three survey types. Following each task, participants completed a three-question survey on their perceived task effort, lower back discomfort and total body discomfort. All questions were presented on a ten-point Numerical Rating Scale (NRS). Following each experimental block, participants completed a survey on their perceived pain using a NRS for Pain [NRS],^40^ and kinesiophobia using four relevant images from the Photograph Series of Daily Activities [PHODA],^41^ which can capture functional deficits when bending and lifting.^42^^,^^43^ At the end of the exosuit block, participants completed an 11-question survey designed to probe general exosuit usability and their likelihood to use an exosuit in three situations using an NRS centered around five to capture a neutral response (Table S1).

### Statistical analysis

The study sample size was estimated on 15 healthy participants lifting a 6 kg mass with a squat and stoop lifting style.^36^ Compared to lifting without an exosuit, lifting with an exosuit reduce peak back extensor EMG amplitudes by 8.3%, around a pooled standard deviation of 11.6% MVIC. Therefore, 14 participants would be required to find significant differences with 80% power with an alpha of 0.05.

Statistical analysis for primary outcome measures was performed using Mixed Effects ANOVAs, which were Bonferroni corrected for pairwise comparisons for co-primary outcome measures. Secondary outcome measures had a conservative alpha (0.01) to prevent type-I error. Significant interaction and main effects were post hoc tested using Tukey’s HSD. These models assume linearity and normality, violations of the assumptions were remedied using transformations suggested by the Johnson’s test in Minitab 19 (Minitab LLC, State College, PA, United States). Within the article, significant conditions (exosuit vs no exosuit) main effects or interactions were the focus of analysis. Additional effects were highlighted in supplemental materials. Given the study’s counterbalanced design, block effects were not considered in our ANOVAs as they represented a combined effect of learning the experimental task, total body fatigue, and potential exosuit adaptation.

Primary outcome measures included Peak EMG amplitudes, analyzed using a five-factor ANOVA included the following factors: i) Condition (2—AS and NS), ii) Muscle Group (3- Back extensor, hip extensor, and trunk flexor), iii) Phase (2—lifting and lowering), and iv) Mass (2– 4 and 6 Kg) and v) Style (2- Squat and Stoop). Peak EMG amplitudes from each muscle site is included in the supplementary materials. A co-primary outcome measure was peak biological back extensor moment which was analyzed using a four-factor ANOVA for i) Condition (2), ii) Phase (2), iii) Mass (2), and v) Style (2).

Secondary biomechanical outcome measures included peak overall hip and lumbar extensor moment and the peak biological lumbar moment using a similar four-factor ANOVA above. Additional secondary outcome measures included kinematics at the torso, hip, knee and ankle compared using a three-factor ANOVA for i) Condition (2), ii) Mass (2), and iii) Style (2). Perceived task effort, low back discomfort, and total body discomfort were analyzed in the same model. Finally, perceptual analysis of post-block LBP and PHODA scores were compared using paired t-tests between conditions. Exosuit usability and likelihood to use were not compared statistically and presented as a qualitative measure.

## Results

### Participant demographics

Fifteen participants volunteered for this study, including 11 women and four men (31 ± 10 years old, 67 ± 11Kg, 169 ± 10cm, and 23 ± 3 m^2^/Kg). The LBP rating for these participants was mild (2.3 ± 1.8), with six participants presenting low pain (< 2/10) on screening. The duration of LBP ranged from 1 to 192 months (49 + 59), with five participants having pain for less than 6 months. For the experimental task, one participant used 80% exosuit assistance when stoop lifting only, finding the application of force to their bottom unsettling; all other participants used 100% assistance. One participant did not complete their 6 kg mass tasks in their final block due to technical difficulties that led to time constraints; however, this participant did complete the post-block survey. No participant experienced adverse events, including skin rashes, chaffing or balance disruptions.

### Biomechanical results

#### General overview

**Figure 3.**
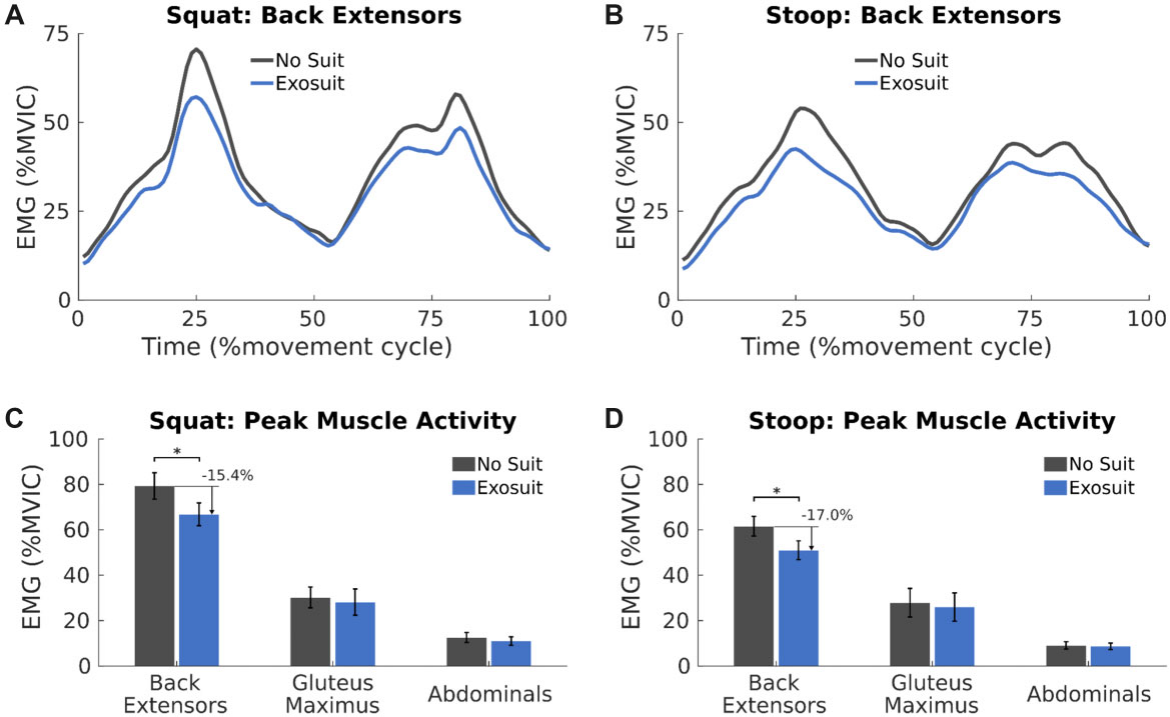
Muscle activation amplitudes of the back extensor (BE) muscles when lifting without (black) and with (blue) an exosuit. Subplot (**A**, **B**) demonstrates the time-varying differences in back extensor muscle activation during a squat (**A**) and stoop (**B**) lift. These differences lead to a reduction in peak EMG amplitudes when squat (**C**) and stoop lifting (**D**). Peak EMG amplitudes of the hip extensors (GM) and abdominals (Abs) were not significantly changed in the exosuit condition (**C**, **D**). Peak back extensor EMG amplitudes are different between lifting styles (**C** > **D**); however, the exosuit achieved a similar reduction in peak muscle activity regardless of lifting style. Significant condition differences within a lifting style are noted (*) and conveyed as a % change from the NS condition. Error bars (**C**, **D**) represent standard error.

**Figure 4.**
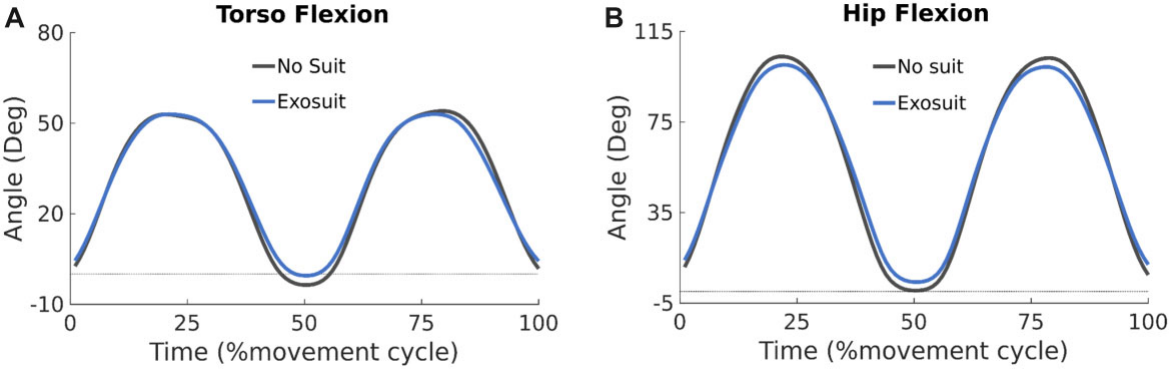
Kinematic comparison between squat lifting with (blue) or without (black) an exosuit, at the torso (**A**), hip (**B**). For all plots, increasing degree is in the direction of flexion with a dashed line indicating if the waveform entered extension.

**Figure 5.**
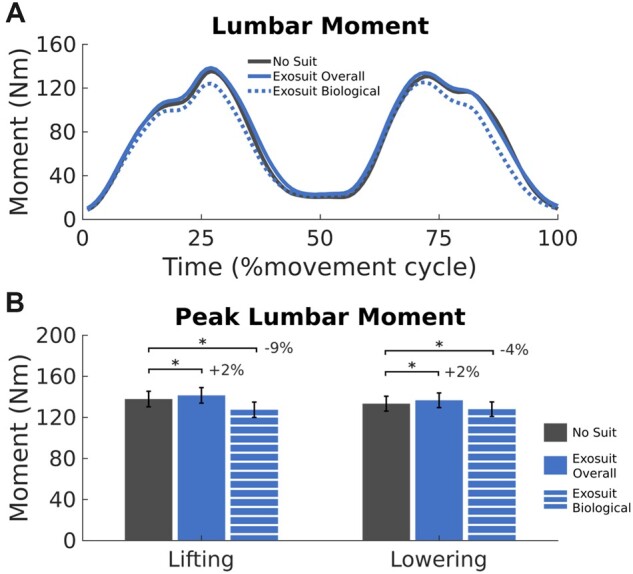
Kinetic comparison between squat lifting without an exosuit (black) and the overall (blue solid) and biological (blue dashed) back extensor moment when lifting and lowering with an exosuit (AS). Subplot (**A**) demonstrates an overall reduction in biological moments across the movement cycle when lifting with an exosuit. Confirmed by a reduction in peak back extensor biological moment particularly when lifting with the exosuit (**B**, left). This exosuit effect was less efficient during the lowering phase of a movement cycle (**A** (50%–100%) and **B**, right). Significant differences between conditions within a lifting phase are noted (*) and conveyed as percentage change from the NS condition. Error bars represent standard error.

For EMG, kinetics, and kinematics, there were main effects for lifting style; capturing biomechanical outcome measures were different between squatting and stooping. However, the back exosuit did not interact with lifting style, and this implies the impact of the back exosuit had similar efficacy across lifting styles (Figure 3). For brevity, Figures 4 and 5 will focus on squat lifting. However, the impact of the exosuit on stooping and the differences between lifting styles can be found in supplementary figures (Figure S1 and S2) and tables.

#### Electromyography

The primary focus of EMG measures was to investigate the influence of the back exosuit on muscle activation amplitudes during lifting. Comparing peak EMG amplitudes identified significant style, phase, condition, and muscle group main effects but no mass main effect (Table S2). A muscle group by condition interaction (*P* < .001) captured for the back extensor muscles lifting with an exosuit reduced peak EMG amplitudes by 16.1% when compared to the no exosuit condition (Figure 3A and B, Table S2). Despite delivering back and hip extensor assistance, the exosuit did not significantly change the peak EMG amplitudes of the gluteus maximus, nor did participants increase peak abdominal EMG coactivity in opposition to these assistive forces (Figure 3C and D, Table S2).

#### Kinematics and kinetics

Secondary biomechanical analysis focused on sagittal plane kinematic and kinetic outcome measures that can confound or corroborate the interpretation of muscle activation amplitudes. Joint kinematics had a significant style main effect (Table S3). There was no mass main effect, and only some kinematic measures were impacted by the exosuit condition (Table S3). At the torso, participants had less (*P* < .001) peak extension (3.1°) at the end of the lifting phase with the exosuit, reducing overall trunk displacement (peak flexion-extension)by 4.7% (Figure 4A, Table S3), however, there was no change in peak torso flexion (Figure 4A, Table S3). The hip also experienced reduced peak extension (3.6° *P* < .001) and peak flexion (3°, *P* < .001), limiting overall hip displacement by 4.1% in extension and 3.4% in flexion (Figure 4B, Table S3). Peak knee and ankle flexion and extension had no significant changes in response to the exosuit condition (Table S3).

Considering joint kinetics, peak back and hip extensor moments experienced significant style, mass, condition and sometimes phase main effects (Table S4). We refer to the overall moment (Figure 5A, solid blue line) as a direct outcome of inverse dynamics. Whereas the biological moment (Figure 5A, dotted blue line) is the overall moment subtracted by the external exosuit moment (see Methods—kinematics and kinetics). Despite reduced peak hip flexion, peak back extensor moments were slightly (3.0 Nm, or 2.3%) yet significantly higher in the exosuit condition when compared to the no-suit condition (*P* < .001, Figure 5B, Table S4). In terms of the biological moment, the peak back extensor moment became significantly lower in the active suit condition than in the no-suit condition. However, this effect was dependent on the phase of the movement (Condition x Phase Interaction, *P* < .001, Table S4), where the exosuit reduced peak biological moment to a greater extent during the lifting phase (10.2Nm or 9.1%) than the lowering phase (5.2Nm or 4.2%) (Figure 5B, Table S4). Analysis of peak hip extensor moments revealed similar features. There was no significant difference in peak overall moments between the exosuit and non-exosuit conditions (Table S4); however, a condition by phase interaction captured peak biological hip extensor moments were reduced when lifting with an exosuit (*P* < .001, Table S4).

### Perceptual results

In addition to the biomechanical benefits of an exosuit, participants had noticeable perceptual benefits. Post-task survey questions captured that participants felt a statistically significant (*P* < .001) reduction in “task effort” (0.3 points on a 10-point scale), and “lower back discomfort” (0.4 points) when they lifted with the exosuit. These benefits were achieved without inducing trade-offs of increased “total body discomfort'' (Table S5). For all measures, there was a mass main effect, and low back effort was considered higher when stoop lifting (Table S5).

**Figure 6.**
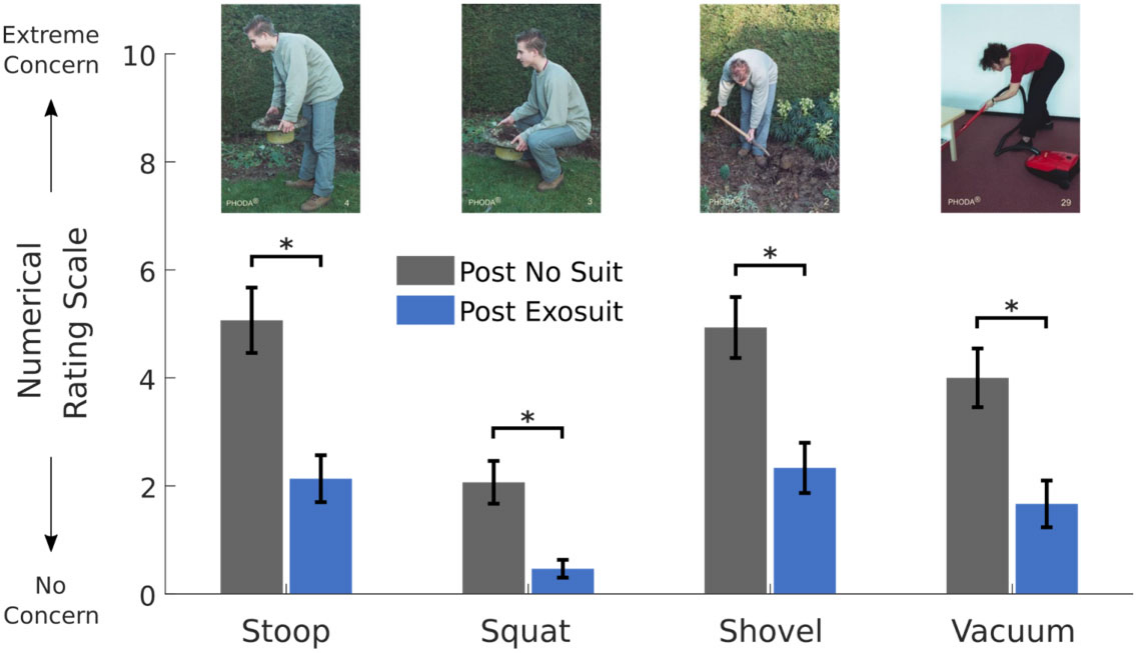
Rating of the participants’ level of concern if they envisioned themselves performing the task displayed in the picture after they performed a block of lifting with (blue) or without (black) the exosuit. Significant differences between conditions are noted (*). Error bars represent standard error.

Following a block of lifting, participants identified a significantly (*P* = .001 or <.001) lower level of concern (range 1.6–3.0) when they pictured themselves performing a series of squatting and stooping activities with the exosuit (Figure 6, Table S6). However, there was no significant difference between the participants’ level of pain after a block of lifting with or without the exosuit (1.7 ± 1.7/10) (Table S5).

#### Exosuit usability and likelihood of usage

**Figure 7.**
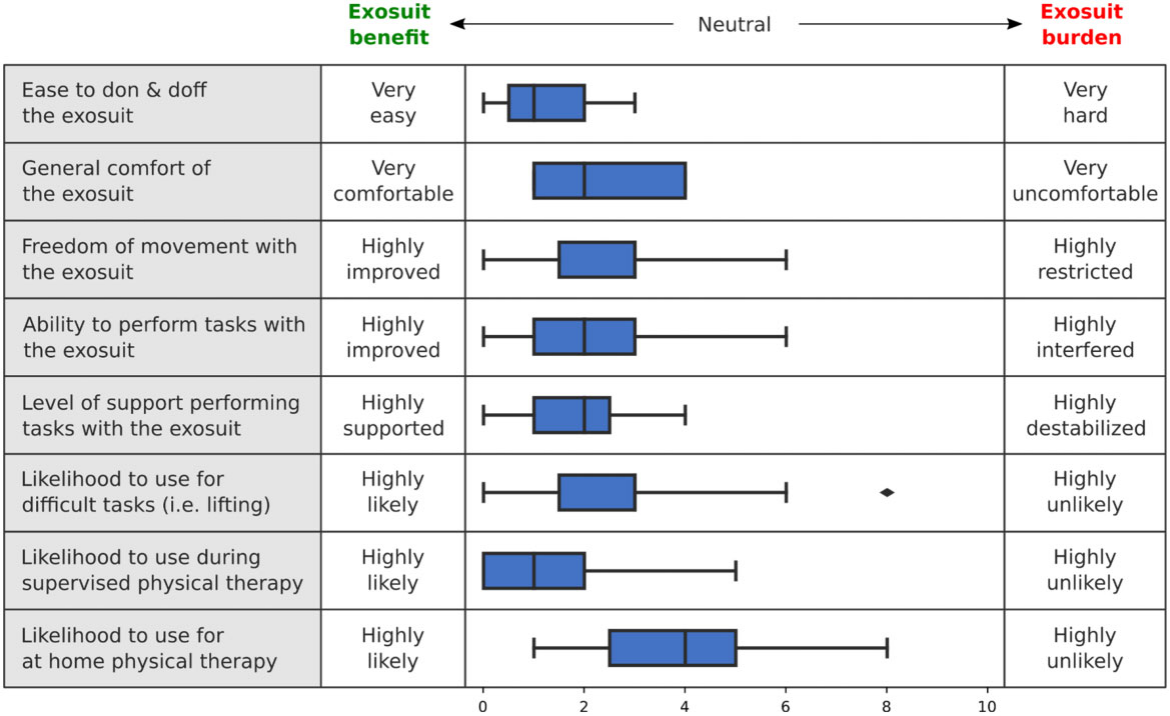
Exosuit usability and potential usage of exosuit on a subset of questions asked in the usability survey. Data are presented as box plots with the median and interquartile range, outliers as a dot.

At the end of the exosuit block, participants appraised exosuit usability and likelihood to use (Table S1). Generally, participants reported good usability in the ease to don and doff, and adjusting the exosuit (Figure 7). Participants reported the overall comfortability of the suit, the ability of the controller to move with them, and the kinematic compatibility to be somewhat good. Only one participant indicated that the exosuit felt as if it made them move unnaturally. Participants also reported the exosuit was generally supportive, good at reducing low back loads, and improving their ability to perform tasks.

The likelihood of using the exosuit appeared to be situational (Figure 7). Participants acknowledged they would be likely to use the exosuit during supervised physical therapy. Participants also indicated they would be somewhat likely to use the exosuit during difficult tasks and during at-home physical therapy; however, 2–3 participants indicated they would not be likely to use the exosuit in this situation (Table S1).

## Discussion

We demonstrated that an active back exosuit provides a biomechanical benefit to participants with LBP. Previous studies have speculated that reductions in back extensor activity, back extensor moments, and perceived task effort may have meaningful benefits to patients during recovery from LBP.^44^ Compared to lifting without an exosuit, lifting with an active exosuit reduced peak biological back extensor moments by 9%, which despite the potential for error in assuming our back exosuit acted around a fixed 0.12 m MAL, is consistent with 8%–12% reduction reported in the literature.^18^^,^^20^ These reductions in peak back extensor moments were complemented by a 16% reduction in peak back extensor EMG amplitudes, consistent with the 10%–30% reduction identified during dynamic lifting tasks.^18^^,^^20^ Interestingly, despite assisting hip extension, we did not see a reduction in gluteus maximus activation. This is consistent with findings we reported in healthy participants performing a constrained lifting task.^36^ However, we found that for a prolonged lifting task, with 320 repetitions, participants did experience a reduction in hip extensor activation amplitudes,^35^ highlighting prolonged or repetitive exosuit use individuals adapt to make these technologies more efficient.^45^

For an exosuit to be considered for use in the rehabilitation of individuals with LBP, it is beneficial to see a biomechanical advantage without restricting natural movements. Our exosuit resulted in small kinematic compensations at the hip and torso during squat and stoop lifting. Peak torso and hip extension, which occurred in the middle (50%) of the movement cycle (Figure 4), was reduced by 4.7%–3.7% compared to no suit, indicating participants adopted a slight postural compensation, possibly to counterbalance the posterior mass of the exosuit.^46^^,^^47^ While peak torso flexion did not change; peak hip flexion was reduced by 3° during the exosuit condition compared to the no-suit condition. While kinematics are seldom reported,^18^ studies generally find a 3–20° reduction in peak trunk flexion when lifting with a back exo,^21–23^^,^^48^^,^^49^ placing our restriction (3°) at the lower end of this spectrum. Across the literature, reduced trunk flexion likely represents how an individual compensates for the impedance delivered by an exosuit, encouraging an individual to move with joints that have low to no assistance.^50^ At a kinetic level, reduced trunk flexion can lead to reduced back extensor moments.^49^ However, our analysis identified peak overall lumbar moment was only slightly (2.7%) or non-significantly higher during the exosuit condition, possibly explained by the mass of the suit.^46^^,^^47^ As a predictor of LBP, any increase in peak back extensor moment should be avoided.^51^ However, once factoring for the assistive forces delivered by the exosuit, peak biological moments were decreased (9.2% and 4.2%) when lifting and lowering with an exosuit suggesting this device does have the potential to limit spinal damage.^52^

Another key concern when adopting exosuit technology in rehabilitation is inducing adverse motor control between wearing the device and not. Previous studies in healthy individuals have identified back exos can increase peak abdominal coactivation for some tasks.^23^ Increased trunk muscle coactivation could increase overall trunk loading,^12^^,^^32^ restrict natural movement patterns,^33^ and potentially exacerbate pain.^34^^,^^53^ Our results demonstrate no difference in peak abdominal EMG amplitudes between suit conditions, indicating exosuit use did not lead to co-activation. Collectively, our biomechanical findings of reduced back extensor EMG amplitudes, reduced back extensor biological moments, minimal changes in trunk kinematics, and no increase in abdominal co-activation would suggest the back exosuit offers the potential to reduce spinal loads if evaluated using an EMG-driven musculoskeletal model.^54^ Therefore, this device could represent a tool to reduce a mechano-nociceptive form of LBP and could provide a stimulus to reduce load-induced tissue inflammation.^4^^,^^55^

A positive finding from this study was that individuals with LBP achieved similar biomechanical efficacy between both squat and stoop lifting styles. Previous studies have shown that assistive forces delivered by back exo can result in users adopting a more squat-like lifting style.^50^^,^^56^ However, this has not been observed with the active exosuit used in this study.^35^ Despite a historical bias toward squat being a safer lifting style, both squat and stoop lifting have unique benefits and are common activities in daily living.^57^ In rehabilitation, patients are often encouraged to adopt variable movement patterns to improve overall function, rather than training one optimal lifting technique.^33^^,^^57^^,^^58^ This opens the possibility for physical therapists to target provocative movements for an individual with LBP, knowing the device would still provide assistance to minimize back exertion regardless of movement task to enable more functional and variable exercises earlier in recovery without setbacks or load-induced flair-ups.^4^

Consistent with our measures of reduced peak back extensor EMG and moments, participants experienced reductions in perceived task effort and back discomfort while using the exosuit. Baltrusch et al., identified using a back exoskeleton for a repetitive 20 kg lifting task could reduce task effort and perceived low back discomfort by 2–3 points.^25^ Given that our device applied one-fifth of the assistance of Baltrusch et al.,^25^ we observe a modest 0.3–0.4 point reduction in these measures (Table S5). Consistent with the small change in low back discomfort, our experiment found that individuals did not experience less back pain following a bout of lifting with an exosuit. It has been demonstrated that wearing a back exoskeleton during a static hold task can reduce back pain once task duration exceeds 1.5 minutes.^27^ Shahvarpour et al. has demonstrated wearing a back belt during a repetitive lifting task can reduce pain in individuals with moderate (4/10) levels of LBP.^16^ The above studies suggest if our sample had higher levels of pain (Table S6), or lifted with more repetitions, we might have observed a significant reduction in pain.

Surprisingly, despite the small reduction in task-specific effort and low back discomfort, a validated PHODA scale demonstrated that participants endorsed lower levels of concern when they envisioned themselves using the exosuit for common squatting and stooping tasks (Figure 6). The specific photos included in this study were selected because they have been associated with functional lifting capacity,^42^^,^^43^ hence this finding might reflect this improved confidence could provide functional benefits. Our finding supports the work of Baltrusch, showing individuals with LBP have higher self-efficacy when lifting with an exoskeleton.^59^ The magnitude of concern reduction measured in this study (1.6–3 point reduction) was larger than what could be achieved by a back belt (1 – 1.5).^16^ The benefit of reducing an individual's level of concern with movement is that fear-avoidance behaviors are conditioned to the anticipation of pain that commonly motivates compensatory movements during daily activities.^8^ However, exosuit-related reductions in the level of concern during activities of daily living may help individuals who have kinesiophobia or fear avoidance behaviors confront fear during rehabilitation.

The collective biomechanical and perceptual benefits of a back exosuit can only be leveraged as a rehabilitation technology if it can achieve suitable usability and user acceptance. Previous research has shown the adoption of back exosuits in both healthy subjects and individuals with LBP is hindered if perceived burdens exceed a user’s perceived benefits.^19^^,^^24^^,^^26^^,^^27^ Common perceived burdens include device discomfort, movement restriction, and general difficulties in donning and using a device.^19^^,^^26^^,^^27^ Qualitative data in this study suggest that participants found our exosuit to have high usability when graded for ease of donning and doffing, ease of adjusting, general comfort and freedom of movement. Many participants acknowledge device benefits including controller compatibility, levels of perceived support, reduction in low back load, and an improved ability to perform tasks (Table S1). These encouraging results are in line with other studies in which individuals with LBP identify benefits toward using back exo technology.^26^^,^^27^ To understand the potential use case for this technology, participants reported they would most like to use the device during formal physical therapy, rating it superior to both at-home use and use during particularly strenuous tasks. As our sample was currently undergoing or recently underwent physical therapy for LBP, this response suggests utility in rehabilitation should be further explored.

Although this pilot study is an important first step, it has limited generalizability. Namely, study participants were recruited from a single physical therapy clinic where members of the study team (G.S. and E.Z.) worked. Although this may have imparted desirability bias, we thought it was necessary to have a rich understanding of patient medical history to ensure safety. Second, due to exosuit familiarization, refamiliarization, and washout, along with the collection of relatively few (20) lifts, this study does not capture how an individual’s biomechanics might adapt to exosuit forces at first exposure and over time.^45^ We also chose to spatially and temporally constrained sagittal plane lifting tasks to limit the confounding influence of movement variability between conditions. Because of this decision, it remains unknown if participants would have modified their movement patterns if this task were not constrained.^50^^,^^56^ Lastly, the study design cannot account for a potential placebo effect. To minimize study burden (added device weight with no assistance), participants did not complete this experiment in a sham exosuit condition. However, future work should consider if the simple act of wearing back exo, without biomechanical benefits, could lead to the perception of reduced task effort, pain, and increased task confidence.^16^

Overall the findings from this study suggest that a soft active back can reduce biomechanical measures of back exertion, reduce perceived task effort and pain, and increase lifting confidence with relatively few perceived burdens to individuals with LBP. Although this study was limited to a single-session experiment using individuals with relatively mild LBP, it does highlight the potential of utilizing the exosuit technology in a therapeutic setting. As a part of the Back Pain Consortium (BACPAC) Research Program, future work will probe the clinical implications of using the technology longitudinally over multiple sessions of physical therapy.^60^

## Conclusion

This article was able to describe a new active lightweight (2.7 kg) back exosuit that was able to reduce measures of back extensor exertion, while inducing only minimal movement restriction, and demonstrating no increase in abdominal muscle coactivation in individuals with LBP for a sagittal lifting task. Despite only using a back exosuit for a short period of time, participants not only identified performing lifting tasks with an exosuit to have lower effort and discomfort than lifting without, these individuals with LBP also projected they would have less concern performing daily lifting and bending tasks if they pictured themselves using a back exosuit. Given this device had high usability across multiple domains and individuals expressed a willingness to use the device during supervised physical therapy, this study represents a pivotal step toward future studies designed to track the longitudinal implications of augmenting physical therapy with a back exosuit as part of BACPAC.

## Supplementary Material

pnad003_Supplementary_Data
