# Supplementary material for: Reducing Back Exertion and Improving Confidence of Individuals with Low Back Pain with a Back Exosuit: A Feasibility Study for Use in BACPAC
Source: Pain Med. 2023 Feb 16;24(Suppl 1):S175–86. doi: 10.1093/pm/pnad003 (PMC10403307; doi:10.1093/pm/pnad003)
Supplement: pnad003_Supplementary_Data [file pnad003_supplementary_data.doc]

*
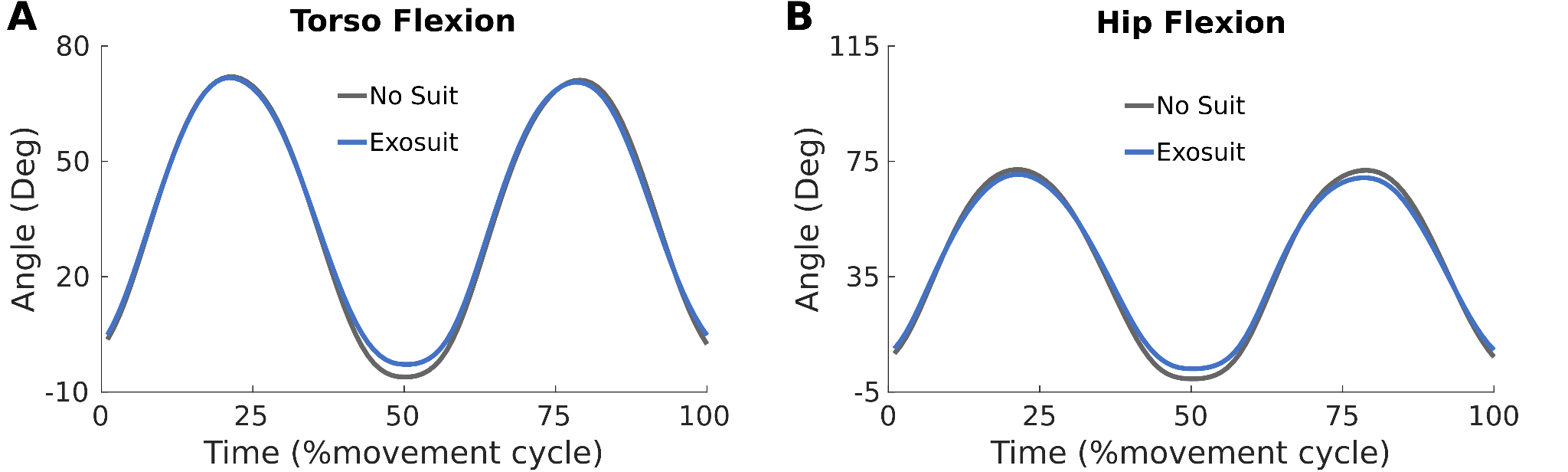
*

**Figure S1:** Kinematic comparison between stoop lifting with (blue) or without (black) an exosuit, at the torso (A), hip (B). For all plots, increasing degree is in the direction of flexion.

*
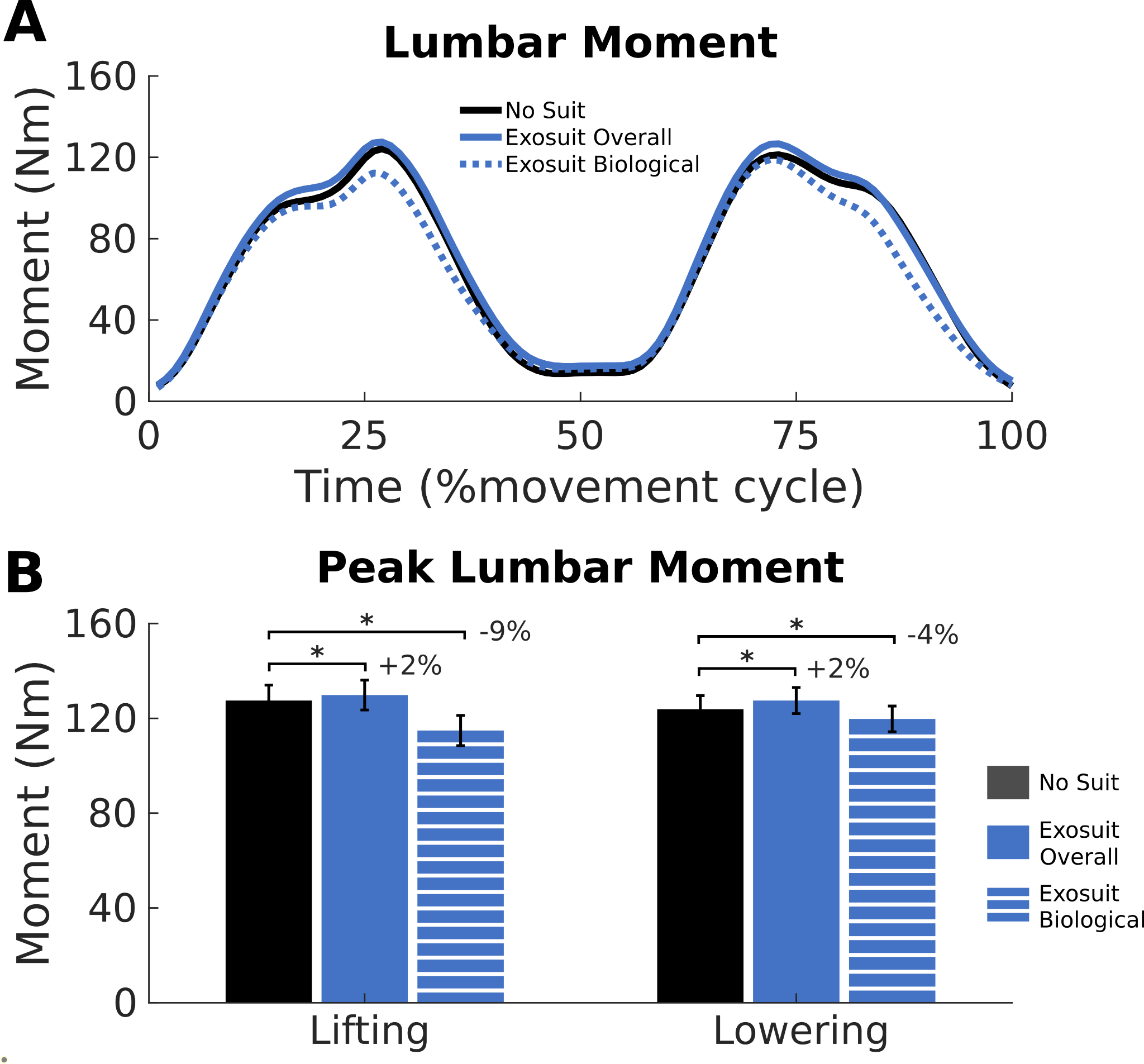
*

**Figure S2:** Kinetic comparison between squat lifting without an exosuit (black) and the overall (blue solid) and biological (blue dashed) back extensor moment when lifting and lowering with an exosuit (AS). Subplot (A) demonstrates an overall reduction in biological moments across the movement cycle when lifting with an exosuit. Confirmed by a reduction in peak back extensor biological moment particularly when lifting with the exosuit (B left). This exosuit effect was less efficient during the lowering phase of a movement cycle (A (50-100%) and B right). Significant differences between conditions within a lifting phase are noted (*) and conveyed as percentage change from the NS condition. Error bars represent standard error

**Table S1**: Exosuit Usability and Likelihood to use, questions and responses

| *Exosuit Usability [Question and Answers]* | | | |
| --- | --- | --- | --- |
|  | Score (Mean (SD)) | N Neutral (5/10) | N Negative (>5/10) |
| *Rate how hard it is to put on and take off the exosuit*  *[Very Easy – Very Hard]* | | | |
|  | 1.4 (1.1) | 0 | 0 |
| *Rate how hard it is to adjust the exosuit*  *[Very Easy- Very Hard]* | | | |
|  | 1.9 (1.3) | 0 | 0 |
| *Rate the general comfort you felt when wearing the exosuit*  *[Very Comfortable- Very Uncomfortable]* | | | |
|  | 2.4 (1.4) | 0 | 0 |
| *Rate how easily/freely you could move when wearing the exosuit*  *[Highly Improved- Highly Restricted]* | | | |
|  | 2.7 (1.6) | 1 | 1 |
| *Rate how well the exosuit predicted and assisted your movements*  *[It moved with me– It forced me to move unnaturally]* | | | |
|  | 2.1 (1.6) | 2 | 0 |
| *Rate the overall ability for you to perform tasks when wearing the exosuit*  *[Highly Improved – Highly Interfered]* | | | |
|  | 2.2 (1.5) | 0 | 1 |
| *Rate the level of support you felt when performing tasks with the exosuit*  *[Highly Supported- Highly Destabilized]* | | | |
|  | 1.9 (1.0) | 0 | 0 |
| *Rate the change in low back load, strain, or effort when performing tasks when wearing the exosuit [Highly Reduced – Highly Increased]* | | | |
|  | 2.2 (1.4) | 1 | 0 |
| *Situational Likelihood to Use an Exosuit* | | | |
| *Rate how likely you would be to use an exosuit for a difficult or hard task like lifting*  *[Highly Likely – Highly Unlikely]* | | | |
|  | 3.2 (2.4) | 0 | 3 |
| *Rate how likely you would be to use an exosuit during supervised exercises or physical therapy*  *[Highly Likely – Highly Unlikely]* | | | |
|  | 1.7 (1.9) | 3 | 0 |
| *Rate how likely you would be to use an exosuit during unsupervised at-home physical therapy exercises [Highly Likely – Highly Unlikely]* | | | |
|  | 3.8 (2.0) | 5 | 2 |

Full questions and the anchor terms of the 10-point scale are presented in italics. Aggregate participant answers are presented as mean and standard deviation. The number of participants with a neutral (5) or below neutral (>5) answer to the question is provided.

**Table S2.** Peak EMG Amplitudes (Mean (SD)) and Statistical Analysis Results for the Lifting task for Muscle Groups

|  | Lifting EMG (% MVIC) | | | Lowering EMG (%MVIC) | | |
| --- | --- | --- | --- | --- | --- | --- |
| Condition | Back Extensors ⸸ | Hip Extensors ⸸ | Abdominals | Back Extensors | Hip Extensors | Abdominals |
| N observations | N=39-42 /45 | N=9-11 /15 | N=25-28 /30 | N=39-42 /45 | N=9-11 /15 | N=25-28 /30 |
| Squat | * | * |  | * | * |  |
| **NS 4Kg** | **76.6 (39.1)** | 28.6 (15.3) | 9.9 (7.6) | **66.0 (31.2)** | 25.3 (15.7) | 9.8 (7.7) |
| AS 4Kg | 67.4 (37.2) | 26.0 (20.6) | 8.7 (6.9) | **58.4 (29.6)** | 24.6 (18.2) | 8.7 (6.8) |
| **NS 6Kg** | **79.3 (41.2)** | 29.5 (16.0) | 10.4 (8.6) | **66.7 (31.8)** | 24.5 (15.8) | 10.7 (9.3) |
| AS 6Kg | 70.5 (37.9) | 31.7 (22.9) | 9.5 (8.0) | **58.5 (27.2)** | 28.4 (25.2) | 9.7 (8.4) |
| Stoop |  |  |  |  |  |  |
| **NS 4Kg** | **61.2 (34.8)** | 25.5 (23.3) | 8.0 (6.3) | **51.5 (25.8)** | 19.9 (16.0) | 7.8 (6.0) |
| AS 4Kg | 53.2 (33.4) | 22.9 (20.1) | 7.9 (5.8) | **48.8 (25.7)** | 20.4 (18.7) | 7.7 (5.6) |
| **NS 6Kg** | **66.1 (35.0)** | 26.0 (21.6) | 8.8 (6.8) | **57.3 (29.9)** | 22.1 (20.7) | 9.0 (6.9) |
| AS 6Kg | 56.7 (30.6) | 24.9 (22.9) | 8.7 (6.6) | **52.0 (25.0)** | 22.8 (19.6) | 8.5 (6.3) |
| Significant Effects | | F Score | Probability |  |  |  |
| Cond | | (1,1182)=4.9 | **0.027** |  |  |  |
| Style | | (1,1182)=19.9 | **<0.001** |  |  |  |
| Mass | | (1,1182)=2.9 | 0.087 |  |  |  |
| M. Group | | (2,1182)=1204.6 | **<0.001** |  |  |  |
| Phase | | (1,1182)=11.6 | **0.001** |  |  |  |
| Cond x M Group | | (2,1182)=5.7 | **0.003** |  |  |  |
| Style x M Group | | (2,1182)=12.7 | **<0.001** |  |  |  |
| Phase x M Group | | (2,1182)=8.2 | **<0.001** |  |  |  |
|  |  |  |  |  |  |  |

Symbols: Significant **condition main effects or interactions are in bold**; symbols capture differences between * style, † mass, ‡ muscle group, and ⸸ phases. Interactions are applied specifically to the muscle group. The symbol is placed on the significantly higher number.

Abbreviations: no exosuit (NS), active exosuit (AS), electromyography (EMG), maximum voluntary isometric contraction (MVIC), condition (Cond), and muscle group (M. Group)

**Table S3.** Peak Angular Displacement (Mean (SD)) Statistical Analysis Results for the Lifting Task Kinematics

|  | Torso (°) | | Hip (°) | | Knee (°) | | Ankle (°) | |
| --- | --- | --- | --- | --- | --- | --- | --- | --- |
|  | Ext. | Flex. | Ext. | Flex. | Ext. | Flex. | Plant. Flex. | Dorsi Flex. |
| Squat |  |  |  | * |  | * | * | * |
| NS 4Kg (N=15) | **3.6 (5.3)** | -54.8 (7.2) | **-0.6 (9.6)** | **-104.5 (10.2)** | 1.0 (5.5) | -96.2 (18.5) | -55.8 (5.4) | -85.7 (6.1) |
| AS 4Kg (N=15) | **0.7 (5.8)** | -53.9 (9.5) | **-4.3 (8.9)** | **-100.2 (9.8)** | 1.0 (5.4) | -97.2 (17.1) | -55.5 (5.0) | -87.4 (5.2) |
| NS 6Kg (N=14) | **4.1 (5.3)** | -55.2 (7.0) | **-0.3 (9.4)** | **-104.4 (10.7)** | 1.9 (4.7) | -97.1 (18.2) | -54.8 (4.6) | -85.7 (5.0) |
| AS 6Kg (N=14) | **0.5 (5.3)** | -54.8 (6.8) | **-4.5 (9.1)** | **-101.6 (10.0)** | 2.2 (5.2) | -96.3 (17.2) | -54.3 (4.3) | -86.6 (4.8) |
| Stoop | * | * | * |  | * |  |  |  |
| NS 4Kg (N=14) | **5.8 (5.2)** | -73.0 (8.5) | **1.0 (8.3)** | **-73.1 (9.6)** | 4.5 (4.8) | -8.7 (7.9) | -45.7 (4.5) | -57.6 (4.1) |
| AS 4Kg (N=14) | **2.1 (5.2)** | -72.5 (8.56) | **-3.1 (7.9)** | **-70.5 (9.1)** | 6.7 (4.2) | -8.3 (12.8) | -44.6 (5.0) | -58.0 (3.6) |
| NS 6Kg (N=13) | **6.1 (4.6)** | -72.8 (7.4) | **0.1 (7.7)** | **-73.3 (10.5)** | 4.9 (4.1) | -7.9 (7.4) | -45.1 (4.6) | -57.2 (4.2) |
| AS 6Kg (N=13) | **3.5 (5.6)** | -72.3 (8.1) | **-2.7 (8.8)** | **-71.3 (9.5)** | 6.2 (3.8) | -9.5 (10.4) | -44.9 (5.5) | -58.0 (3.8) |
| Significant Main Effects or Interactions | | | | | | | | |
| Cond (p) | **<0.001** | 0.562 | **<0.001** | **0.032** | 0.07 | 0.882 | 0.397 | 0.099 |
| Style (p) | **<0.001** | **<0.001** | **0.034** | **<0.001** | **<0.001** | **<0.001** | **<0.001** | **<0.001** |
| Mass (p) | 0.707 | 0.993 | 0.497 | 0.819 | 0.759 | 0.909 | 0.810 | 0.906 |
| Cond (F) | (1,90)=63.7 | (1,90)=0.34 | (1,90)=59.4 | (1,90)=4.7 | (1,90)=3.4 | (1,90)=0.02 | (1,90)=0.7 | (1,90)=2.8 |
| Style (F) | (1,90)=19.2 | (1,90)=305.9 | (1,90)=4.6 | (1,90)=519.3 | (1,90)=40.2 | (1,90)=1322.5 | (1,90)=245.1 | (1,90)=2632.4 |
| Mass (F) | (1,90)=0.1 | (1,90)=0.0 | (1,90)=0.5 | (1,90)=0.1 | (1,90)=0.1 | (1,90)=0.0 | (1,90)=0.1 | 1,90)=0.0 |

Symbols: Significant **condition main effects or interactions are in bold**; symbols capture differences between * style, and † mass. The symbol is placed on the significantly higher number. F Scores convey degrees of freedom

Abbreviations: no exosuit (NS), active exosuit (AS), condition (Cond).

**Table S4.** Peak Moments (Mean+SD) and Statistical Analysis Results for the Lifting Task

|  | Lumbar Ext. Overall | | | Lumbar Ext. Biological | | | | Hip Ext. Overall | | Hip Ext. Biological | | |
| --- | --- | --- | --- | --- | --- | --- | --- | --- | --- | --- | --- | --- |
| Condition | Lift (Nm) | Lower (Nm) | | Lift (Nm) | | Lower (Nm) | | Lift (Nm) | Lower (Nm) | Lift (Nm) | | Lower (Nm) |
| Squat | *⸸ | * | | * | | * | | *⸸ | * | * | | * |
| NS 4Kg (N=15) | 134.8 (28.7) | 129.2 (26.7) | | **134.8 (28.7)⸸** | | **129.2 (26.7)** | | 148.4 (21.9) | 141.9 (21.5) | **148.4 (21.9)⸸** | | **141.9 (21.5)** |
| AS 4Kg (N=15) | **137.6 (29.8)** | **133.1 (28.3)** | | 123.6 (29.2) | | 124.1 (27.5)⸸ | | 146.5 (20.7) | 140.3 (21.6) | 129.2 (19.8) | | 128.6 (20.7)⸸ |
| NS 6Kg (N=14) | 142.9 (29.9)† | 138.3 (28.8)† | | **142.9 (29.9)†⸸** | | **138.3 (28.8)†** | | 157.3 (23.0)† | 151.9 (21.9)† | **157.3 (23.0)†⸸** | | **151.9 (21.9)†** |
| AS 6Kg (N=14) | **147.6 (29.3)**† | **141.8 (26.9)**† | | 133.5 (29.2)† | | 133.2 (26.5)†⸸ | | 157.4 (20.8)† | 150.1 (18.4)† | 139.9 (20.6)† | | 139.1 (17.9)†⸸ |
| Stoop | ⸸ |  | |  | |  | | ⸸ |  |  | |  |
| NS 4Kg (N=14) | 127.1 (30.2) | 122.9 (26.2) | | **127.1 (30.2)**⸸ | | **112.9 (26.2)** | | 135.5 (25.3) | 130.5 (22.7) | **135.5 (25.3)⸸** | | **130.5 (22.7)** |
| AS 4Kg (N=14) | **129.8 (28.1)** | **126.1 (24.6)** | | 114.9 (28.5) | | 118.1 (23.9)⸸ | | 136.3 (21.4) | 131.1 (18.4) | 117.9 (21.7) | | 120.6 (16.8)⸸ |
| NS 6Kg (N=13) | 132.5 (24.7)† | 128.7 (22.9)† | | **132.5 (24.7)**†⸸ | | **128.7 (22.9)†** | | 141.5 (20.3)† | 136.8 (18.9)† | **141.5 (20.3)†⸸** | | **136.8 (18.9)†** |
| AS 6Kg (N=13) | **133.9 (24.4)**† | **132.3 (21.1)**† | | 118.8 (24.6)† | | 124.6 (20.8)†⸸ | | 140.9 (18.4)† | 138.1 (15.3)† | 122.2 (18.4)† | | 128.2 (15.0)†⸸ |
| Sig. Effects | F Score | Probability | | F Score | | Probability | | F Score | Probability | F Score | | Probability |
| Cond | (1,194)=15.9 | **<0.001** | | (1,194)=100.6 | | **<0.001** | | (1,194)=0.2 | 0.68 | (1,194)=255.6 | | **<0.001** |
| Style | (1,194)=57.3 | **<0.001** | | (1,194)=57.4 | | **<0.001** | | (1,194)=136.1 | **<0.001** | (1,194)=139.9 | | **<0.001** |
| Mass | (1,194)=148.2 | **<0.001** | | (1,194)=144.0 | | **<0.001** | | (1,194)=113.3 | **<0.001** | (1,194)=113.6 | | **<0.001** |
| Phase | (1,194)=27.2 | **<0.001** | | (1,194)=1.9 | | 0.172 | | (1,194)=33.9 | **<0.001** | (1,194)=3.9 | | 0.05 |
| Cond*Phase | (1,194)=0.693 | 0.727 | | (1,194)=17.4 | | **<0.001** | | (1,194)=0.0 | 0.957 | (1,194)=15.45 | | **<0.001** |
|  |  | |  | |  | |  |  |  |  |  | |

Symbols: Significant **condition main effects or interactions are in bold**; symbols capture differences between * style, † mass, and ⸸ phases. Interactions are applied specifically to the muscle group. The symbol is placed on the significantly higher number.

Abbreviations: no exosuit (NS), active exosuit (AS), extension (Ext.), condition (Cond)

**Table S5.** Numerical Rating Scale for Task Difficulty and Discomfort (Mean+SD) and Statistical Analysis for the Lifting Task.

|  | Task Difficulty | Low Back Discomfort | Total Body Discomfort |
| --- | --- | --- | --- |
| Squat |  |  |  |
| NS 4Kg (N=15) | **1.5 (0.9)** | **1.3 (1.1)** | 1.2 (1.1) |
| AS 4Kg (N=15) | 1.2 (1.1) | 0.7 (1.1) | 1.3 (1.2) |
| NS 6Kg (N=14) | **2.3 (1.2)**† | **1.3 (1.4)**† | 1.7 (1.4)† |
| AS 6Kg (N=14) | 1.9 (1.1)† | 1.3 (1.2)† | 1.5 (1.3)† |
| Stoop |  | * |  |
| NS 4Kg (N=15) | **1.5 (1.0)** | **1.5 (1.2)** | 1.5 (1.4) |
| AS 4Kg (N=15) | 1.4 (1.1) | 1.0 (1.4) | 1.4 (1.4) |
| NS 6Kg (N=14) | **2.6 (1.2)**† | **1.8 (1.6)**† | 1.8 (1.6)† |
| AS 6Kg (N=14) | 2.0 (1.5)† | 1.4 (1.4)† | 1.9 (1.5)† |
| Significant Main Effects or Interactions | | | |
| Cond (p) | **0.002** | **0.008** | 0.643 |
| Style (p) | 0.2 | **0.039** | 0.091 |
| Mass (p) | **<0.001** | **<0.001** | **0.009** |
| Cond (F) | (1,96)=9.7 | (1,96)=7.3 | (1,96)=0.2 |
| Style (F) | (1,96)=1.7 | (1,96)=4.4 | (1,96)=2.9 |
| Mass (F) | (1,96)=41.8 | (1,96)=6.5 | (1,96)=7.0 |

Symbols: Significant **condition main effects or interactions are in bold**; symbols capture differences between * style, and † mass. Interactions are applied specifically to the muscle group. The symbol is placed on the significantly higher number.

Abbreviations: no exosuit (NS), active exosuit (AS), extension (Ext.), condition (Cond)

**Table S6.** Numerical Rating Scales for Pain and PHODA (Mean + SD) and significant effects

|  | Pain | Photograph Series of Daily Activities (PHODA) | | | |
| --- | --- | --- | --- | --- | --- |
| Condition |  | Stoop | Squat | Shovel | Vacuum |
|  |  |  |  |  |  |
| NS | 2.0 (1.7) | **5.1 (2.3)** | **2.1 (1.5)** | **4.9 (2.2)** | **4.0 (2.1)** |
| AS | 1.5 (1.6) | 2.1 (1.7) | 0.5 (0.6) | 2.3 (1.8) | 1.7 (1.7) |
| Sig. Effects |  |  |  |  |  |
| Probability | 0.177 | <0.001 | **0.001** | <0.001 | <0.001 |

Symbols: Significant **condition main effects or interactions are in bold**; All scales are scored from 0-10.
